# Identification of pBC218/pBC210 Genes of *Bacillus cereus* G9241 in Five Florida Soils Using qPCR

**DOI:** 10.1155/2014/197234

**Published:** 2014-07-03

**Authors:** Vicki Ann Luna, Kimmy Nguyen, Damian H. Gilling

**Affiliations:** Center for Biological Defense, College of Public Health, University of South Florida, Tampa, FL 33612, USA

## Abstract

The distribution of the virulent plasmid pBC210 of *B. cereus* that carries several *B. anthracis* genes and has been implicated in lethal anthrax-like pulmonary disease is unknown. We screened our collection of 103 *B. cereus* isolates and 256 soil samples using a quantitative PCR (qPCR) assay that targeted three open reading frames putatively unique to pBC210. When tested with DNA from 2 *B. cereus* strains carrying pBC210, and 64 Gram-positive and 55 Gram-negative bacterial species, the assay had 100% sensitivity and specificity. None of the DNA from the *B. cereus* isolates yielded positive amplicons but DNA extracted from five soils collected in Florida gave positive results for all three target sequences of pBC210. While screening confirms that pBC210 is uncommon in *B. cereus*, this study is the first to report that pBC210 is present in Florida soils. This study improves our knowledge of the distribution of pBC210 in soils and, of public health importance, the potential threat of *B. cereus* isolates carrying the toxin-carrying plasmid. We demonstrated that sequences of pBC210 can be found in a larger geographical area than previously thought and that finding more *B. cereus* carrying the virulent plasmid is a possibility in the future.

## 1. Introduction

The ubiquitous bacterium* Bacillus cereus* found in soils, fresh and marine waters, dust, and intestinal systems of many insects has usually been linked to food poisoning yet has also caused a variety of serious infections [1–5]. Historically, it has been proposed that* B. cereus* can acquire one or both* B. anthracis* megaplasmids (pXO1 and pXO2) and currently it is reported that* B. cereus* carries similar plasmids (i.e., pBCXO1) and subsequently is able to cause anthrax-like illnesses [1, 6, 7]. The megaplasmid pBCXO1 may have been acquired from* B. anthracis* or evolved after acquisition from some distant ancestor. Some unusual cases of* B. cereus* infections were fatal anthrax-like pneumonia in immunocompetent healthy workers [8–10]. An isolate associated with these cases,* B. cereus *G9241 and related strain 03BB87, carried two large plasmids responsible for their virulence [9, 11, 12]. One plasmid pBCXO1 with >99% similarity to pXO1 carried the genes encoding for toxins, while the second pBC210 (formerly pBC218) appeared to carry the genes that encode for a putative polysaccharide (non-D-glutamyl polypeptide) capsule involved with evasion of host immune responses [9, 11, 13–15]. At 210 kb, the pBC210 is smaller than originally calculated and has been referred to as either pBC218 or pBC210 in the literature [16]. We will use the latter designation. Studies have shown that both plasmids have to be present for G9241 to achieve full virulence [6].

Some researchers debate whether* B. cereus* G9241 should be considered a frank or opportunistic pathogen [6]. The fact that* B. cereus *G9241 contains two virulent megaplasmids places it with the pathogenic* B. anthracis*. However, animal studies have equated the virulence of* B. cereus* G9241 to that of* B. anthracis* Sterne as opposed to the highly virulent* B. anthracis* Ames [6]. Yet healthy individuals have died from infection with* B. cereus *G9241. Therefore, it is important to determine the distribution of the pBC210 plasmid and any* B. cereus *strains carrying it. In order to screen our soil sample and* Bacillus* collection, we developed a rapid quantitative PCR (qPCR) assay to target three open reading frames that have been reported as only in pBC210 and so discriminate* B. cereus* with pBC210 from closely related bacillus and other bacteria. We screened DNA extracts of 256 soil samples collected from Florida, Texas, and elsewhere in the United States and DNA from 103* B. cereus* and 119 other bacterial strains (64 Gram-positive and 55 Gram-negative) obtained from clinical and environmental (soils, powders, water, and environmental swab) samples held in our bacterial collection.

## 2. Methods and Materials

### 2.1. Soil Samples

The Center for Biological Defense (CBD) has a collection of DNA extractions procured from 256 soils collected in Florida, Texas, and elsewhere. Many were collected by CBD in Florida, while other soils were obtained from Dr. Rick Zartman at Texas Tech University, Lubbock, TX, and Dr. Dale Griffin at U.S. Geological Survey, Coastal and Marine Science Center, St. Petersburg, FL. DNA had been extracted from the soils as previously reported and tested for the* B. anthracis* toxin and capsule genes as reported elsewhere [17–20]. All DNA had been stored at −30°C until used.

### 2.2. Bacterial Strains

We examined 153* Bacillus* strains (one* B. cereus* G9241, one* B. cereus* G9241-derived, 13* B. anthracis*, one* B. badius*, 103* B. cereus*, five* B. marisflavi*, five* B. megaterium*, five* B. mycoides*, three* B. pseudomycoides*, five* B. pumilus*, five* B. subtilis*, and six* B. thuringiensis*) (Table 1). In addition, we examined 16 other Gram-positive bacterial strains (four genera) (two* Enterococcus faecalis*, one* Lactobacillus rhamnosus*, 12* Staphylococcus aureus*, and one* Streptococcus pneumoniae*) and 55 Gram-negative bacterial strains covering 14 genera (Table 1).

The two* B. cereus *G9241 strains (CBD 1056 and CBD 1057) were given to CBD by the Hoffmaster laboratory, while the other* B. cereus* bacterial strains were obtained from the American Type Culture (ATCC, Manassas, VA, USA) or isolated from soils, powders, swab specimens, and marine samples collected in Florida, Texas, and elsewhere.* B. anthracis*,* Burkholderia mallei,* and* Burkholderia pseudomallei* strains were received from either the CDC or the NIH Biodefense and Emerging Infections Research Resources Repository (BEI Resources, Bethesda, MD, USA). All other bacteria studied were procured from ATCC, the Florida Department of Health, Bureau of Laboratories, Tampa, FL (FDOH), from Mote Marine Laboratory, Sarasota, FL, or from the University of Washington Medical Center in Seattle, WA.

Manipulations of all bacterial strains except for* B. anthracis*,* B. cereus* G9241,* B. mallei,* and* B. pseudomallei* were performed in a BSL2 laboratory in a biological safety cabinet following safety practices outlined in the* Biosafety in Microbiological and Biomedical Laboratories*, 5th Edition (BMBL). All manipulations of cultures of* B. anthracis, B. cereus* G9241,* B. mallei,* and* B. pseudomallei* strains were performed in a biological safety cabinet in a biological safety level 3 (BSL3) laboratory. All safety protocols followed the BMBL practices for the BSL3 environment including the use of protective laboratory clothing and respiratory equipment (US DHHS, 2007). The safety and security requirements by US federal regulation DHHS 42 CFR 73 were strictly adhered to.

### 2.3. DNA Extraction from Bacterial Isolates

Bacteria were grown on tryptic soy agar supplemented with 5% sheep red blood cells (blood agar (BA)) (Remel, Lenexa, KS, USA). All culture plates were incubated at 35°C overnight (18–24 hours) before performing DNA extractions. Following manufacturers' instructions, all genomic DNA extractions were performed using the Epicentre (Qiagen Inc., Madison, WI) extraction kits, a MagNaPure Compact automated instrument (Roche, Inc., Indianapolis, IN, USA), or a boil preparation method used by the Tampa FDOH, a reference lab in the Laboratory Response Network, and is described as follows. Bacterial growth from an overnight cultured media plate was removed and placed into 100 *μ*L of sterile water and boiled for 5 minutes, placed onto ice for 2 minutes, and centrifuged at 12,000 ×g for 10 min at 4°C. The supernatant was transferred to a 0.1 *μ*M filter tube (Millipore Corporation Billerica, MA) and centrifuged for 2 min at 8,000 ×g. 10 *μ*L of all filtrates and DNA extractions of BSL3 isolates (following the University of South Florida Institutional Biosafety Committee guidelines) were used to inoculate a BA media culture plate and incubated at 35°C for 2 days. Extracts and boil preparation filtrates having no growth were allowed out of the BSL3 environment and made available for molecular work. All DNA were stored at 4°C or −30°C until used.

### 2.4. Plasmid Extraction of pBC210

Extractions of pBC210 from the two* B. cereus* G9241 strains (CBD 1056 and 1057) were performed as previously described [21]. The extracts were tested for sterility as the genomic DNA above and stored at 4°C or −30°C until used.

### 2.5. qPCR Design

Open reading frames (ORFs) that according to other researchers are specific to pBC210, pBC218-0047, and pBC218-0072 (formerly pBC218-0073) were selected as targets for primer design [11]. The whole gene sequence for each ORF was identified on the GenBank sequence for pBC210 (accession number AAEK01000004.1). For the internal amplification control (IAC), the 16S rRNA sequence of* B. cereus* (GenBank accession number X55060) was used. These sequences were then copied and placed into the online program PrimerQuest by Integrated DNA Technologies (IDT) (Coraville, IA) to design the real-time PCR primers and probes (Table 2). Parameters necessary for optimal primer and probe design were that the amplicon should be 50–150 bp, the primer melting temperatures close to 50–60°C, and probe melting temperature 10°C higher (68–70°C). Confirmation of pBC210 plasmid in the positive soil DNA extracts was performed using a primer set designed specifically to ORF BC218-0067 that encodes for a transcriptional regulator protein LytR on the pBC210 GenBank sequence (accession number AAEK01000004.1) (Table 2). Later, this primer set was used to test all of the bacterial and soil sample DNA extracts (Table 1).

The qPCR reaction mixture (final volume, 20 *μ*L) consisted of 3 *μ*L of DNA template (ideally 0.5–1.0 ng/*μ*L) and 10 *μ*L TaqMan Fast Universal PCR Master Mix 2X (Applied Biosystems, Foster City, CA). The volumes of the primer and probe working stocks (100 *μ*M) were 0.09 *μ*L of each primer and 0.03 *μ*L of probe, giving final concentrations in the reaction of 450 nM (primer) and 150 nM (probe). The remaining 6.79 *μ*L of the reaction mixture consisted of molecular biology grade water (Fisher Scientific, Fair Lawn, NJ). All qPCR was performed on the ABI 7500 Fast Real Time PCR system (ABI, Delray Beach, FL) with the following conditions: initial Taq activation at 95°C for 20 seconds followed by 40 cycles of 95°C for 3 seconds and 60°C for 30 seconds.

### 2.6. Limit of Detection, Sensitivity, and Specificity of qPCR

To be confident our assay was robust and could detect the pBC210 sequence targets, we determined its limit of detection (LOD), sensitivity and specificity. For the LOD, the concentration (ng/*μ*L) of both genomic and plasmid DNA from the two strains of* B. cereus* G9241 (CBD 1056 and CBD 1057) was measured using the GeneQuant* Pro* spectrophotometer (Biochrom Ltd., Cambridge, England) and then diluted 10-fold from 10^−1^ to 10^−9^ in two separate series and used as template for the qPCR assays as above using the different primer sets. All dilution samples were tested in duplicate in an assay and all assays were performed at least twice. All three target sequence assays were examined. CT values from 30 to 35 were considered positive, 20–30 were strong positives, and 36–39 were weak positives. A dilution that yielded CT values greater than 40 was considered too weak, and the limit of detection would correspond to the concentration of DNA in the preceding dilution.

Although only two isolates of* B. cereus* G9241 were available, the sensitivity of the assay primer sets was determined by multiple testing (250 tests) of these strains using the optimal concentration (0.75 ± 0.25 ng/*μ*L) of DNA as template. Specificity was determined by testing the assay primer sets against 48 isolates of* Bacillus *species, including 27 isolates of closely related members of the* B. cereus *group (*B. anthracis, B. mycoides, B. pseudomycoides,* and* B. thuringiensis*), and against both Gram-positive and Gram-negative bacterial isolates listed on Table 1. The presence of pBC210 in* B. cereus* isolates and in the environment was determined by testing the assay against the 103* B. cereus* in our collection (isolated from clinical and environmental sources) and against DNA extracted from 256 soils previously tested for* B. anthracis* (Table 1).

## 3. Results and Discussion

The qPCR assay targets and identifies the presence of three open reading frames reported by other researchers to be exclusively in the pBC210 plasmid [10]. All qPCR assays for the targets consistently gave positive results in over 250 tests with DNA from the two positive control strains (CBD 1056 and 1057) (Table 1). The LOD for the targets in the assay was 0.06471 ng/*μ*L for CBD 1056 and 0.06873 ng/*μ*L for CBD 1057 using genomic DNA and three logs lower for plasmid DNA (Table 3). For simplicity, Table 3 gives the values of only one target sequence (ORF 0047) but all three sequences were examined and yielded essentially the same LOD for both positive control strains. All tests using DNA from other bacteria, including many isolates in the* B. cereus* group (excluding other* B. cereus*), produced negative qPCR results. Because the assay had 100% sensitivity and specificity, we were confident that it would detect the pBC210 plasmid in our soil samples. When we tested the DNA extracted from the 256 soils that had been previously tested for* B. anthracis* plasmids, pXO1 and pXO2, we found five (2%) soil samples that produced positive qPCR amplicons for ORF 0047 and ORF 0072 (Table 1). These five soil samples were all from Florida and also yielded positive amplicons for ORF 0067 (Table 4). Upon repeated testing all five soil samples consistently produced positive amplicons for all three targets. None of these five soils produced a positive amplicon for only one but not the other targets. In previous tests, three of the samples gave negative results for pXO1 and pXO2, while two soils produced positive qPCR amplicons for pXO1 (Table 4). None of the other soils from Florida or elsewhere produced any positive amplicons for any one of the three target sequences of pBC210. As expected, the CT values for the positive controls for the three sequences were much smaller when plasmid was used as template as opposed to that obtained from genomic DNA template (Table 1 legend). The CT values for the target sequences obtained from the positive control genomic DNA extractions (Table 1 legend) were closer to but not quite as high as the CT values obtained from tests of the positive soils (Table 4). This suggests a much lower number of bacteria with the target genes being present in the soils. The fact that the CT values vary from soil to soil sample and even from one target assay to another may reflect the low number of bacteria or that the target genes are not of the same numbers in each sample or are an artifact of sampling error when the testing was performed. It is very interesting that the testing of soil sample 42 produced much lower CT values for ORF 0067 than for ORF 0047 and ORF 72. This could have been due to a testing method problem or the fact that ORF 0067 was present in the sample in much higher copies, perhaps also in other plasmids, or in other bacteria.

We then screened the DNA from the 103* B. cereus* isolates in our collection. These had not been previously examined for pBC210 by other means and so were unknowns. Therefore, data using these strains were not included in specificity calculations. None of the DNA from the 103* B. cereus* isolates produced positive amplicons for the pBC210 targets (Table 1) or for pXO1 or pXO2. Most of these isolates came from soils, powders, clinical samples, and marine samples that were collected in various parts of the United States, but primarily from Texas and Florida.

This assay appears to identify the* B. cereus* G9241 from closely related* Bacillus* spp. and other bacteria with 100% specificity. The assay is based on specific sequences of pBC210 putatively not found on other known* Bacillus* plasmids [11]. It is theoretically possible that one target or the other could be carried by a currently unknown plasmid but using two putatively unique targets on pBC210 lowers the assay's potential for false positive results. The possibility of false positives is further reduced by the use of the third DNA target also reportedly unique to pBC210. Yet the possibility still exists that the three sequences are on different plasmids or chromosome of more than one bacterial cell in the soil.

We are the first to find evidence of three DNA sequences reportedly unique to pBC210 in Florida soils. Even though only 256 soils were tested, we still identified five soils that yielded positive qPCR for the three pBC210 targets. This represents 2% of all of the soils tested and 7% of the Florida soils. This work illustrates the need to survey many more soil samples to better understand the prevalence of the targeted genes, the pBC210 plasmid, and virulent* B. cereus *like* B. cereus* G9241 in the environment. At the time of initial testing of the soils, our laboratory was solely looking for* B. anthracis* and not for* B. cereus *and no isolates of* B. cereus* from those first tests were saved for further testing. Although many attempts were made using different selective media and methods, we were unable to isolate any bacterial strains that carried the plasmid from the scant amount we had left of the five positive soil samples. We surmise that the number of pBC210 carrying* B. cereus* may be extremely low when compared to other* B. cereus *strains in each soil sample. Yet the fact that we identified five soils that produced positive qPCR results for 3 target genes that are putatively unique to pBC210 demonstrates the need to test more soils and to isolate the* B. cereus* or other bacterial isolates that may carry the target genes on either the plasmid or the chromosome.

The primary obstacle in evaluating* B. cereus* isolated from patient cultures is overcoming the commonly held view that* B*.* cereus* is an “insignificant laboratory contaminant” [1]. While few* B. cereus* derived pulmonary infections have proven to be lethal, pBC210 has been found to be carried by the* B. cereus *isolated from some of these lethal cases. The fact that our study shows that the virulent plasmid may be found in more areas than in the Texas/Louisiana areas gives strength to the argument that clinicians might consider the possibility of a virulent* B. cereus *as the cause of infection. Future screening of more soils across Florida, Texas, Louisiana, and other states would give a clearer picture of the geographical distribution of this plasmid. In addition, the search would ideally include examining other* Bacillus* species besides* B. cereus* because none of our soils that were positive for the plasmid yielded* B. cereus* isolates that carried the plasmid.

## 4. Conclusion

This study is the first to report that three sequences reportedly unique to the pBC210 plasmid are present in DNA extractions from Florida soils. We suggest that the distribution of the pBC210 plasmid be expanded from Texas and Louisiana to now include Florida. We were able to identify five soil samples that appear to have the pBC210 plasmid present using qPCR targeting three open reading frames that are putatively only found in pBC210. These five samples are 2% of the total number of soils tested but represent 7% of Florida soils. This higher percentage may indicate that* B. cereus* or other* Bacillus* spp. carrying the virulent plasmid are more prevalent in Florida than in Texas or Louisiana. Thus, further work to examine the distribution of pBC210 carrying* Bacillus* sp. not only in Florida but also in other areas is warranted.

## Figures and Tables

**Table 1:** Results of the qPCR assay targeting ORF 0047 and ORF 0072 unique to pBC210 using bacterial and soil DNA extracts as template.

DNA source (*N*)	PCR assay		
	ORF 0047	ORF 0072	ORF 0067
**Bacteria (244)**			
* B. cereus* G9241 (2)	+	+	+
**Other *Bacillus* species (151)**			
* B. cereus* (103)	−	−	−
* B. anthracis* (13)	−	−	−
* Bacillus badius *(1)	−	−	−
* B. marisflavi* (5)	−	−	−
* B. megaterium* (5)	−	−	−
* B. mycoides* (5)	−	−	−
* B. pseudomycoides* (3)	−	−	−
* B. pumilus* (5)	−	−	−
* B. subtilis* (5)	−	−	−
* B. thuringiensis *(6)	−	−	−
**Other bacteria (71)**			
**Gram-positive (16)**			
* Enterococcus faecalis *(2)	−	−	−
* Lactobacillus rhamnosus *(1)	−	−	−
* Staphylococcus aureus *(12)	−	−	−
* Streptococcus pneumoniae *(1)	−	−	−
**Gram-negative (55)**			
* Achromobacter xylosoxidans *(1)	−	−	−
* Acinetobacter baumannii *(1)	−	−	−
* Acinetobacter calcoaceticus *(1)	−	−	−
* Burkholderia cepacia *(15)	−	−	−
* Burkholderia mallei *(5)	−	−	−
* Burkholderia pseudomallei* (5)	−	−	−
* Burkholderia *species* *(6)	−	−	−
* Cedecea neteri *(1)	−	−	−
* Citrobacter freundii *(1)	−	−	−
* Enterobacter cloacae * (1)	−	−	−
* Escherichia coli* (6)	−	−	−
* Klebsiella pneumoniae *(1)	−	−	−
* Pseudomonas aeruginosa* (1)	−	−	−
* Salmonella enterica* (6)	−	−	−
* Serratia marcescens *(1)	−	−	−
* Shigella flexneri *(1)	−	−	−
* Stenotrophomonas maltophilia *(1)	−	−	−
* Vibrio parahaemolyticus *(1)	−	−	−
**Soils from United States (256)**			
Florida (69)	5+, 64−	5+, 64−	5+, 64−
Texas (72)	−	−	−
Elsewhere (115)	−	−	−

^a^“+” denotes a positive amplicon that was produced by the assay with a cycle threshold (CT) value of ≤39.99. For ORF 0047, the average CT for the two controls was 16.66 with a range of 15.92–18.73 using plasmid DNA and 29.21 with a range of 20.93–38.25 using genomic DNA. For ORF 0072, the average CT for the two controls was 16.10 with a range of 15.24–18.03 for plasmid DNA and 27.09 with a range of 21.29–33.60 using genomic DNA. All samples positive for the two ORF sequences were also positive for ORF 0067. For ORF 0067, the average CT for the two controls was 15.24 (range of 12.25–15.91) using plasmid DNA and 30.72 (range of 28.59–33.99) using genomic DNA. For the 16S assay, the average CT for the two positive controls was 20.40 with a range of 13.68 to 27.20 for genomic DNA. For all DNA extractions of both bacterial and soil samples, the average CT value was 21.24 and the range was from 12.89 to 39.97. The two *B. cereus* carrying pBC210 (CBD 1056 and CBD 1057) were tested a minimum of 250 times.

^b^“−” denotes a negative result as an amplicon was not detected in the qPCR assay. All samples including the five Florida soils that produced positive results for the target sequences (ORF 0047, ORF 0067, and ORF 0072) were tested in duplicate in multiple runs by two different personnel.

**Table 2:** Oligonucleotide primers and probes used in qPCR assays.

ORF on pBC210	Protein or gene	Primer	Sequence (5′→3′)	Location^a^	Amplicon length (bp)	Source
0047	Electron transport protein Rv1937	47F	GGTGATTCGATCAGGTTCATTA	54385–54406	140	This study
		47R	CGCCATCCCGGTATTAAAG	54506–54524		
		47P^b^	TTTGAATCGACAGCAGCCTGCTGA	54430–54453		

0067^c^	Transcriptional regulator	67F	CAATTGCCTTTAATACGTCACC	82127–82148	135	This study
		67R	CTCGTATGCGTTATGAAGACC	82241–82261		
		67P^b^	TGACGTTGCCGTATTTGACGACCGA	82204–82228		

0072^d^	Polysaccharide polymerase	72F	ACCCTAGTCCTTTCCCAAATA	87419–87439	111	This study
		72R	GCACAAACCAACAAGGAGA	87511–87529		
		72P^b^	TGCCAAGCTTCTTCCCTTCCAGAAAGA	87472–87498		

	16S rRNA	16F	GCGGTGGAGCATGTGGTT	948–965	73	This study
		16R	AGGGTTTTCAGAGGATGTCAAGAC	997–1020		
		16P^b^	AATTCGAAGCAACGCGAAGAACCTTACCA	967–995		

^a^The location of oligonucleotide sequences was determined using the pBC210 plasmid sequence of *B. cereus* G9241 (GenBank accession number AAEK01000004.1) for ORF 0047, ORF 0067, and ORF 0072. For the internal amplification control, the *B. cereus* 16S rRNA sequence (GenBank accession number X55060) was used.

^b^For direct PCR amplicon detection during the reaction, all probe oligonucleotides were labeled with a 6-FAM (6-carboxy-flourescein) reporter at the 5′ end and a Black Hole Quencher-1 quencher at the 3′ end.

^c^The qPCR assay for ORF 0067 was used to confirm all positive tests determined by assays for ORF 0047 and ORF 0072 and was later performed on all of the bacterial and soil sample DNA.

^d^ORF 0072 is previously notated as pBC218-ORF 0073 (Hoffmaster et al., 2006) [11], while GenBank currently designates it as ORF 0072.

**Table 3:** The limit of detection of assay for ORF 0047 using genomic and plasmid DNA extracts from *B. cereus* G9241 (CBD 1056).

DNA concentration (ng/*μ*L)		CT^a^ value average (range)	
Genomic	Plasmid	Genomic DNA	Plasmid DNA
647.1	17.5	24.68 (24.63–24.73)	14.15 (14.14–14.18)
64.71	1.75	24.12 (24.04–24.18)	15.23 (15.12–15.36)
6.471	0.175	27.00 (26.95–27.03)	17.24 (17.64–17.89)
0.6471	1.75 × 10^−3^	30.71 (30.57–31.02)	20.82 (20.69–21.95)
0.06471	1.75 × 10^−4^	36.35 (35.89–36.58)	24.96 (24.80–25.11)
6.471 × 10^−3^	1.75 × 10^−5^	38.97 (38.92–40.00)	28.41 (28.35–28.54)
6.471 × 10^−4^	1.75 × 10^−6^	Undetected^b^	32.63 (31.58–33.77)
6.471 × 10^−5^	1.75 × 10^−7^	Undetected	34.87 (34.51–35.29)
6.471 × 10^−6^	1.75 × 10^−8^	Undetected	37.03 (36.48–39.57)
6.471 × 10^−7^	1.75 × 10^−9^	Undetected	Undetected

The genomic DNA was extracted using the boil preparation method, while plasmid DNA was extracted as previously described [17]. The starting DNA concentration of CBD 1057 was 687.3 ng/*μ*L and 16.5 ng/*μ*L for genomic and plasmid extractions, respectively. The CT averages and ranges for ORF 0067 and ORF 0072 were similar (±1.2 and ±1.4, respectively, for genomic DNA) to the values above for both CBD 1056 and 1057. Thus, these were not included in this table. CT values ≥40 are regarded as negatives. The limit of detection was then determined to be the prior dilution.

^a^“CT” denotes cycle threshold value.

^b^“Undetected” denotes that no CT value was given for the sample tested.

**Table 4:** Average CT^a^ value (and CT ranges) produced by DNA extracts of the five Florida soil samples positive for pBC210.

Sample number	CT value (range) for assay target ORF^b^ of pBC210			*B. anthracis* plasmids^c^	
	ORF 0047	ORF 0072	ORF 0067	pX01	pX02
7	37.21 (36.36–38.44)	36.39 (35.55–37.34)	36.43 (36.43–36.43)	−	−
8	37.67 (36.79–38.80)	37.15 (35.39–38.73)	36.00 (35.93–36.67)	−	−
14	38.44 (37.13–38.15)	36.52 (36.18–37.50)	36.25 (35.42–36.08)	−	−
39	35.91 (35.46–37.29)	35.16 (34.36–38.35)	34.99 (34.47–35.52)	+	−
42	38.84 (36.89–38.87)	36.51 (33.71–38.57)	28.81 (28.59–29.05)	+	−

^a^“CT” denotes cycle threshold value.

^b^The five soils obtained from Florida yielded positive results for ORF 0047 and ORF 0072. Subsequently, the soils were also tested with the primer and probe set for ORF 0067 for confirmation. All assays were performed in duplicate in multiple runs and by two different personnel in order to rule out errors.

^c^Soils had been previously tested for the genes *lef, pag, * and *cya* of pX01 and *capC* on pX02 of *B. anthracis* as previously reported [16–18].
